# Explaining Correlates of Cervical Cancer Screening among Minority Women in the United States

**DOI:** 10.3390/pharmacy10010030

**Published:** 2022-02-15

**Authors:** Manoj Sharma, Kavita Batra, Christopher Johansen, Siddharth Raich

**Affiliations:** 1 Department of Social and Behavioral Health, School of Public Health, University of Nevada, Las Vegas, NV 89119, USA; manoj.sharma@unlv.edu (M.S.); raichs2@unlv.nevada.edu (S.R.); 2 Office of Research, Kirk Kerkorian School of Medicine, University of Nevada, Las Vegas, NV 89102, USA; kavita.batra@unlv.edu

**Keywords:** Multi-theory Model, cervical cancer, screening, minority women

## Abstract

Globally, cervical cancer is the fourth leading cause of death among women. While overall cervical cancer rates have decreased over the last few decades, minority women continue to be disproportionately affected compared to White women. Given the paucity of theory-based interventions to promote Pap smear tests among minority women, this cross-sectional study attempts to examine the correlates of cervical cancer screening by Pap test using the Multi-theory Model (MTM) as a theoretical paradigm among minority women in the United States (U.S.). Structural Equation Modelling (SEM) was done for testing the construct validity of the survey instrument. Data were analyzed through bivariate and multivariate tests. In a sample of 364 minority women, nearly 31% (*n =* 112) of women reported not having received a Pap test within the past three years compared to the national rate (20.8%) for all women. The MTM constructs of participatory dialogue, behavioral confidence, and changes in the physical environment explained a substantial proportion of variance (49.5%) in starting the behavior of getting Pap tests, while the constructs of emotional transformation, practice for change, and changes in the social environment, along with lack of health insurance and annual household income of less than $25,000, significantly explained the variance (73.6%) of the likelihood to sustain the Pap test behavior of getting it every three years. Among those who have had a Pap smear (*n* = 252), healthcare insurance, emotional transformation, practice for change, and changes in the social environment predicted nearly 83.3% of the variance in sustaining Pap smear test uptake behavior (adjusted R^2^ = 0.833, F = 45.254, *p* < 0.001). This study validates the need for health promotion interventions based on MTM to be implemented to address the disparities of lower cervical cancer screenings among minority women.

## 1. Introduction

Cervical cancer is the fourth leading cause of death in the world among women [1]. It was estimated that, worldwide, 570,000 women were diagnosed with cervical cancer and approximately 311,000 women died due to cervical cancer in 2018 [1]. While there has been a decrease in cervical cancer mortality in the United States (U.S.), approximately 13,800 women are diagnosed and approximately 4290 women die per year [2,3]. These rates have decreased over the past few decades, yet cervical cancer disparities in the U.S. continue to affect minority women, (i.e., women who are not of White/European heritage such as African American, Hispanic/Latino, Asian American, Pacific Islander, American Indian, etc.) [4].

The Papanicolaou (Pap) test is an effective cervical cancer screening tool to decrease the rates of cervical cancer [5]. The American Cancer Society (ACS) recommends cervical cancer screening with an HPV test alone every 5 years for everyone with a cervix from age 25 until age 65 [6]. Rates of cervical cancer screening (Pap testing) in the U.S. were 81.7% for women 21–44 years old and 79.2% from women 45–64 years old [7], but the rates of cervical cancer screening are lower for Pap testing among minorities in the U.S. and disparities still exist for African Americans, Hispanics/Latina, Asians, and American Indians compared to White women [2,3,8,9,10,11]. 

Cervical cancer occurs primarily in low-resource, underserved areas and is typically associated with poverty, race/ethnicity, and/or other health disparities [3,11,12,13,14]. Outside the U.S., researchers found that in Norway, immigrant women reported lower adherence to cervical cancer uptake compared to native Norwegian women [15]. Similar associations have been found among Non-European Union and European Union migrants compared to German women [16], Middle Eastern women from Asian and Middle-Eastern countries [17], and Syrian refugees in Greece [18]. West African migrant women reported lower knowledge related to the importance of cervical cancer screening [19] and in China, uptake of cervical cancer screening services in Chinese migrant workers is lower than non-migrant workers in China [20].

Two determinants of cervical cancer incidence are carcinogenic HPV infection and lack of access to cervical cancer screening [3,12]. Other possible important correlates of cervical cancer screening include lack of adequate access to preventive services and not utilizing these services (e.g., lack of transportation, fear of results, and mistrust of the health care system) [12,15,16,17,18,19,20,21,22,23,24,25,26,27].

Interventions to increase Pap testing among minority women have had some success and, in some cases, self-sampling for HPV has performed well [28,29,30,31]. For example, a culturally tailored randomized control trial (RCT) cervical cancer screening intervention among Latinas reported that women in the intervention arm had increased Pap test screening compared to those in the control group [32]. Another culturally tailored RCT intervention among North American Chinese women reported that women in the intervention arms (i.e., community outreach or direct mail) increased Pap test screening compared to women in the control group, suggesting that culturally and linguistically appropriate interventions may increase Pap test levels [33]. Another study among African American women reported that African immigrant women reported lower knowledge of cervical cancer and lower Pap test screening rates compared to African American women [34]. Yet, more studies focusing specifically on minority women in the U.S. are needed to promote Pap testing. 

Few interventions among minority women utilized health behavior theories. For example, in a review, Brevik (2020) found that four RCTs of culturally tailored intervention materials were associated with a 54% increase in Pap testing [32,35,36,37,38]. However, many of these studies did not use theory in their interventions. Those that did use theory drew from social cognitive theory, the health belief model, the transtheoretical model, the social support model, elaboration theory, or multiple theories [39,40,41,42]. In sum, there are limited studies to increase cervical cancer screening among minority women and only a few of these interventions among minority women utilized theory. 

There is a need to focus on newer models, such as the fourth-generation Multi-theory Model (MTM) of health behavior, to explain correlates of cervical cancer screening among minority women in the U.S. The MTM has been used in previous health behavior studies, such as for COVID-19, sleep, HPV vaccination, mammography, and melanoma, but to date, researchers have not tested the MTM on cervical cancer screening [43,44,45,46,47,48]. The purpose of this study was to examine the correlates of cervical cancer screening by Pap test using MTM as a theoretical paradigm in U.S. minority women.

## 2. Materials and Methods

### 2.1. Study Design, Setting, and Sampling

Data for this cross-sectional, descriptive, and U.S. based study were collected from 7 October to 12 October through actively managed, double-opt-in market panels recruited by the Qualtrics team. The Qualtrics utilize high-quality research panels and quota sampling to meet the specific requirements of the sample requested by researchers. Previous literature described the differences between traditional survey and market-based or commercial research panels [35]. As described by Qualtrics (more information available at https://www.qualtrics.com/research-services/online-sample/, accessed on 10 February 2022), the company uses multiple strategies (e.g., dynamic surveys in a dashboard style, app-based recruitment, or through online/mobile games and social media) to recruit eligible participants through convenience sampling. Respondents can self-select to participate in the survey if they satisfy the eligibility criteria. Given the use of multiple avenues, response rate is difficult to compute. The recruitment of the sample is based on the quota constraints and screening opted by researchers who signed a contractual agreement with Qualtrics. In addition, Qualtrics ensures the quality of data by checking for bots, duplicates, speeders, and fraudulent responses before providing a complete and high-quality dataset to the researchers. Participants could be screened out due to multiple reasons: (1) if they did not qualify for the inclusion criteria; (2) if the quota was already filled during fielding; and (3) if participants took significantly less time (less than half the median time) to complete the survey, which would indicate lack of thoughtfulness to answer the questions.

### 2.2. Participants’ Selection Criteria

Women belonging to racial minority groups, aged between 21 and 65 years, living in the U.S., who had the ability to understand the English language, and provided voluntary informed consent were eligible to participate in this study. If participants opted to take the survey, they were asked to answer a few screening questions without revealing the original objective of the study. This was done to prevent self-selection and response bias. Eligible participants who thoughtfully completed the survey were compensated through incentives per terms and conditions set forth by Qualtrics and its data collection partners. 

### 2.3. Ethical Considerations

The study (protocol #1804208-1) was deemed “exempt” by the institutional review board in accordance with the Federal regulatory statutes. Detailed information about study’s objective and significance was provided in an information sheet, which helped participants to make informed decisions about participating in the study. In other words, participation in this survey was completely voluntary. All methods were performed in accordance with the relevant guidelines and regulations.

### 2.4. Data Integrity

Qualtrics used multiple ways, such as digital fingerprinting and ‘prevent ballot box stuffing option’, to prevent multiple responses from the same participant. No identifying information was collected during the survey and responses were anonymized to prevent the collection of IP address, location data, and contact information. Data were provided to the researchers in an encrypted file. 

### 2.5. Survey Tool

**Figure 1:**
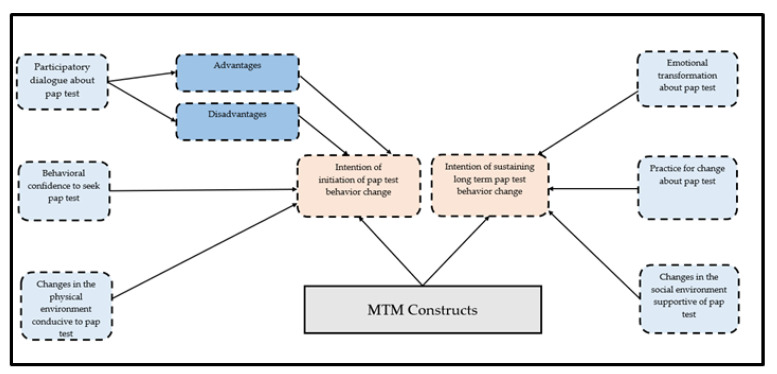
MTM theoretical framework for a survey tool used to explain Pap test behavior among racial minority groups.

A 48-item questionnaire including 18 items related to sociodemographic factors and the remaining 30 items related to MTM constructs was developed based on the fourth-generation behavioral theory proposed by Sharma and Petosa in 2014 [36]. The MTM offered a robust approach to explain several health-related behaviors in the past [29,30,31,32,33,34]. This tool to explain the correlates of the Pap test underwent a series of iterations to check its face and content validity by a group of Subject Matter Experts (SMEs). Experts in behavioral theories, health promotion, and cancer-related research areas were randomly selected to participate in a blinded review of the tool. Their feedback and comments were addressed in a series of revisions before the final version of the tool was obtained. Once finalized, this tool was assessed for its construct validity using structural equation modelling described in detail in the methodology section. For the initiation model, except “changes in physical environment” (measured by 2 items), the subscales of perceived advantages, perceived disadvantages, and behavioral confidence (surety to overcome external and internal barriers) were measured by 5 items measured on a 5-point Likert scale. The “advantages” and “disadvantages” were measured on a frequency scale which included the following response options: never (0), almost never (1), sometimes (2), fairly often (3), and very often (4). Participatory dialogue was a difference derivative of “advantages” and “disadvantages”, and the score could range from −20 units to +20 units. For “change in physical environment” and “behavioral confidence”, a scale of surety was used which ranged from “not at all sure” to “completely sure.” For sustenance, “emotional transformation” and “practice for change” scales were measured on 3 items each. However, “changes in social environment” was measured through 5 items. Emotional transformation is the ability to transform emotional distress into a positive emotional state. Practice of change entails actions to maintain the behavior initiated despite the challenges and “changes in social environment” considers the role of the social support system (family and friends) to help change a particular behavior. A detailed description of the components (initiation and sustenance) of the survey is provided in Figure 1.

### 2.6. Minimum Sample Size Calculation

Referring to the G*power 3.1.9.7 software (linear multiple regression: fixed model, R^2^ increase), a minimal number of 154 participants was required to reach significance when considering the following statistical parameters: type I error α = 5%, power 1-β = 95%, a moderate effect size f^2^ = 0.15, and a total number of variables *N* = 15 to be integrated in the multivariable regression analysis [37,38]. Given the lack of consensus in the sample size recommendations for the Structural Equation Modelling (SEM), we used Kline (2015) criteria that had 20 observations (participants) for each estimated parameter in the model, with a typical size of *N* = 200, among models using the maximum likelihood method [39]. However, recently, several studies recommended a sample size which would vary from 50 to 400 participants [40,41,42]. Our study sample meets the minimum requirements to yield hypothesized effects.

### 2.7. Data Analyses

The SPSS software v.26 (Armonk, NY, USA: IBM Corp.) was used to conduct the descriptive and inferential statistical analysis. All assumptions were tested prior to the application of statistical models. Comparison between groups for the normally distributed numeric data was conducted using the independent samples test, whereas the chi-squared test was used to compare categorical data among groups. The Pearson correlation test was used to correlate two continuous variables. Hierarchical regression was conducted by taking initiation and sustenance scores as dependent variables. Polytomous variables used in the regression were dummy-coded. The statistical significance was denoted as *p* < 0.05. Missing data analysis was not warranted as a complete dataset and was obtained from Qualtrics.

The SPSS AMOS software v.24 [43] was utilized to perform the Structural Equation Modelling (SEM) for testing the MTM model. All items of the main constructs of the MTM instrument were used as indicators of the latent variables of initiation and sustenance (described elsewhere in the text). A fair pair of items with similar contents were allowed correlation measurement errors. The maximum likelihood method was used for estimation. Multiple indices of goodness-of-fit were used: the relative chi-square (χ2/df; cut-off values: <2–5), the Root Mean Square Error of Approximation (RMSEA; close and acceptable fit are considered for values <0.05 and <0.11, respectively), the Tucker Lewis Index (TLI), and the Comparative Fit Index (CFI; acceptable values are ≥0.90) [44,45]. For each model, the overall fit, significance of structural paths, and amount of variability of the latent variables accounted for by the observed variables were assessed [39,45,46]. Standardized estimates for path coefficients, interpreted as regression coefficients, were calculated for all proposed relationships in the model. Reliability diagnostics were also performed.

## 3. Results

In a sample of a total of 364 participants, two hundred and fifty-two (69.2%) participants reported having the Pap smear test and nearly 31% had not had the Pap test over the past 3 years (Table 1). Among those who had a Pap smear test, the majority of the participants had it normally. The median age of both groups were comparable. The majority of the respondents from both groups were non-Hispanics, Christians, and African Americans, and had comorbidities of non-psychological in origin (Table 1). Upon comparing the socio-economic and healthcare access factors, participants who had not had a Pap smear test were less educated (5.4% vs. 0.8%; *p* < 0.001), uninsured (28.6% vs. 5.2%; *p* < 0.01), and unemployed (59.8% vs. 38.9%; *p* < 0.001), and had a lower income (27.7% vs. 19.8%; *p* = 0.004; Table 2). Among the participants with no Pap smear test, only 31.3% reported being recommended by their healthcare providers for a Pap test as opposed to the 58.3% of participants with a Pap test (Table 2). As indicated in Table 3, participants with a Pap test had higher overall initiation (3.02 ± 0.99 vs. 1.69 ± 1.41; *p* < 0.001) and sustenance mean scores (2.98 ± 1.06 vs. 1.50 ± 1.34; *p* < 0.001; Table 3) as opposed to those who had not had a Pap smear test in the past. Except for the subscale “perceived disadvantages”, the mean scores of “perceived advantages”, “participatory dialogue”, “behavioral confidence”, and “changes in physical environment” were significantly higher among participants who had a Pap smear test in the past than those who had not had a Pap smear test (Table 3). The mean scores of “perceived disadvantages” were not statistically different among both groups. For sustenance, the scores of all the subscales, namely “emotional transformation”, “practice for change”, and “changes in social environment”, were higher among participants with a Pap smear test, with statistically significant mean differences compared to the participants who had not had a Pap smear test in the past (Table 3).

Table 4 shows the Pearson correlation coefficient matrix of all the observed variables. Perceived advantages are directly correlated with perceived disadvantages (r = 0.26, *p* < 0.01), behavioral confidence (r = 0.58, *p* < 0.01), changes in physical environment (r = 0.50, *p* < 0.01), emotional transformation (r = 0.47, *p* < 0.01), practice for change (r = 0.46, *p* < 0.01), and changes in social environment (r = 0.49, *p* < 0.01). Changes in physical environment is strongly and directly correlated with behavioral confidence (r = 0.75, *p* < 0.01), emotional transformation (r = 0.73, *p* < 0.01), and changes in social environment (r = 0.64, *p* < 0.01). Changes in social environment is directly correlated with practice for change (r = 0.82, *p* < 0.01) and emotional transformation (r = 0.78, *p* < 0.01, Table 4). The reliability of the entire scale was 0.94, with individual scales’ reliability ranging from 0.81 to 0.94 (Table 4).

**Figure 2:**
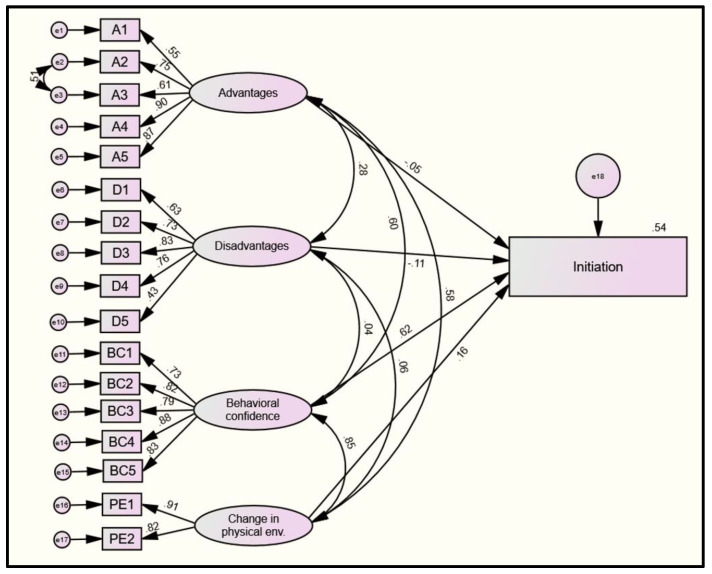
Model diagram of the four-factor model of the initiation. Legend: e1–17 = error terms 1–17; A = advantages; D = disadvantages; BC = behavioral confidence; and PE = change in the physical environment. Latent variables/factors are represented with ovals. Measured/manifest variables are represented with squares. Single-headed arrows indicate a hypothesized direct relationship between two variables. Double-headed arrows demonstrate the bi-directional relationship (i.e., covariance).

**Figure 3:**
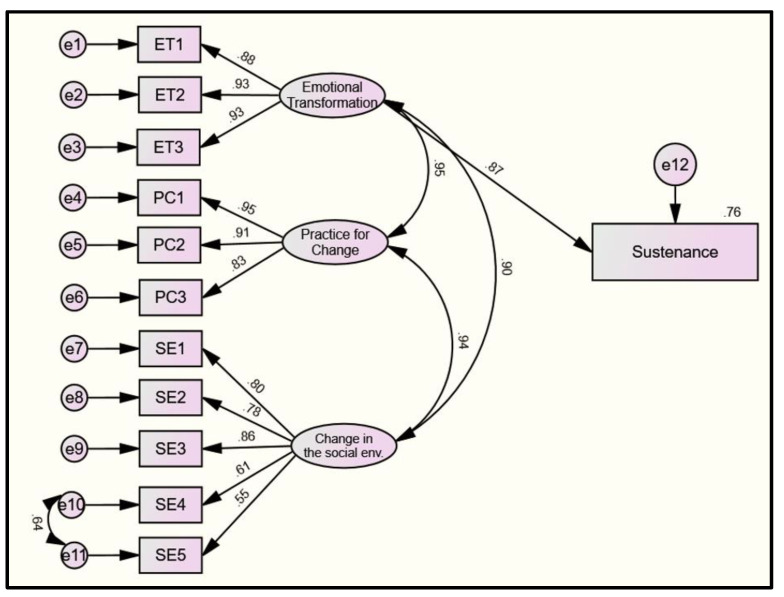
Model diagram of the three-factor model of the sustenance. Legend: e1–11 = error terms 1–11; ET = emotional transformation; PC = practice for change; BC = behavioral confidence; and SE = change in the social environment. Latent variables/factors are represented with ovals. Measured/manifest variables are represented with squares. Single-headed arrows indicate a hypothesized direct relationship between two variables. Double-headed arrows demonstrate the bi-directional relationship (i.e., covariance).

All goodness-of-fit indices suggest that the presented models (Figure 2 and Figure 3) reasonably fit the data. For the initiation model, the relative chi-square (χ2/df = 2.51; cut-off values: <2–5), the Root Mean Square Error of Approximation (RMSEA = 0.064 (LCL 0.056; UCL 0.073); close and acceptable fit are considered for values <0.05 and <0.11, respectively), the Tucker Lewis Index (TLI = 0.94), and the Comparative Fit Index (CFI = 0.95; acceptable values are ≥0.90) were reasonable to indicate model fit. The estimates of each structural relationship between the MTM subscales and initiation are shown in Figure 2. Upon observing the standardized effects of latent variables on factor loadings, statistically significant effects that ranged from moderate to large were found. Except for “perceived disadvantages”, all other latent variables, including “perceived advantages” (β = 0.55 to 0.90), “behavioral confidence” (β =0.73 to 0.88), and “changes in physical environment” (β = 0.82 to 0.91), had large effects on their reflective indicators, which is suggestive of the valid measurements of the constructs used (Figure 2). Behavioral confidence had a moderate direct effect on initiating Pap smear behavior (β = 0.62, *p* < 0.001), whereas disadvantages had a small direct negative effect (β = −0.11, *p* = 0.02). Effects of advantages and change in physical activity were insignificant. 

Similarly, for the sustenance model, the relative chi-square (χ2/df = 2.44), the Root Mean Square Error of Approximation (RMSEA = 0.063 (LCL 0.049; UCL 0.077)), the Tucker Lewis Index (TLI = 0.97), and the Comparative Fit Index (CFI = 0.98) were calculated. The estimates of each structural relationship between the MTM subscales and sustenance are shown in Figure 3. All latent variables, including “emotional transformation” (β = 0.88 to 0.93), “practice for change” (β = 0.83 to 0.95), and “changes in social environment” (β = 0.55 to 0.86), had moderate–large effects on their reflective indicators, which is suggestive of the valid measurements of the constructs used (Figure 3). Emotional transformation had a large direct effect on the sustenance of Pap smear behavior (β = 0.87, *p* < 0.001), as shown Figure 3.

In a multilevel regression model of initiation, Model 4 (final model) predicted nearly 50% of the variance in initiating Pap smear test uptake behavior among participants who had not had it over the past 3 years (adjusted R^2^ = 0.495, F = 30.66, *p* < 0.001, Table 5). With each unit increment in the subscales of initiation (i.e., participatory dialogue, behavior confidence, and changes in physical environment), the conditional mean for initiating Pap smear uptake behavior increased by 0.021, 0.117, and 0.106 units, respectively (Model 4, Table 5). None of the slopes of socio-economic and healthcare access variables were significant, which indicates no significant differences in the conditional mean changes in initiating Pap smear test uptake behavior among participants who had not had this test done over the past 3 years. 

In a multilevel regression model of sustenance, Model 4 (final model) predicted nearly 74% of the variance in initiating Pap smear test uptake behavior among participants who had not had it before (adjusted R^2^ = 0.736, F = 85.338, *p* < 0.001, Table 6). With each unit increment in the subscales of sustenance (i.e., emotional transformation, practice for change, and changes in social environment), the conditional mean for sustaining Pap smear uptake behavior increased by 0.184, 0.097, and 0.032 units, respectively (Model 4, Table 6). Except for healthcare insurance and lower income, none of the slopes of socio-economic and healthcare access variables were significant, which indicates no significant differences in the conditional mean changes in the sustenance of Pap smear test uptake behavior among participants who had not had this test done over the past 3 years. 

In a multilevel regression model of sustenance among those who had a Pap smear (*n =* 252), Model 4 (final model) predicted nearly 83.3% of the variance in sustaining Pap smear test uptake behavior (adjusted R^2^ = 0.833, F = 45.254, *p* < 0.001, Table 7). With each unit increment in the subscales of sustenance (i.e., emotional transformation, practice for change, and changes in social environment), the conditional mean for sustaining Pap smear uptake behavior increased by 0.168, 0.111, and 0.032 units, respectively (Model 4, Table 7). Except for healthcare insurance, none of the slopes of socio-economic and healthcare access variables were significant, which indicates no significant differences in the conditional mean changes in the sustenance of Pap smear test uptake behavior among participants who had this test done over the past 3 years. 

## 4. Discussion

The study aimed to utilize the contemporary Multi-theory Model (MTM) of health behavior change to explain the determinants of cervical cancer in minority women in the U.S. In our sample, 30.8% of the minority women had not received a Pap test within the past three years, which is much higher than the national statistics for all women of 20.8% [7,24]. This finding points at the continued disparities for Pap test screening among minority women in comparison to their White counterparts.

In our study, all three constructs of MTM, namely participatory dialogue (advantages of getting cervical cancer screening outweighing the disadvantages), behavioral confidence (futuristic surety emanating from self, powerful others, Almighty, etc.), and changes in the physical environment (support from the surroundings), were significantly associated with the intent to initiate cervical cancer screening among women who had not received a Pap test over the past three years and together they accounted for 49.5% of the variance in explaining the dependent variable. This is a substantial proportion of the variance in behavioral and social sciences [49]. Likewise, all three constructs of MTM, namely emotional transformation (directing emotions into goals), practice for change (persistent reflection on behavior change), and changes in the social environment (support from family, friends, etc.), as well as lack of health insurance and annual household income less of than $25,000, significantly explained 73.6% of the variance in the likelihood to sustain the Pap test behavior of getting it every three years. 

The results of our study align with the previous literature as well as they provide newer insights into the correlates for developing interventions to promote cervical cancer screening through Pap tests. The finding in this study that poverty (annual household income of less than $25,000) and lack of insurance are barriers to cervical cancer screening among minority women is supported by the previous literature [12,13,14,24,26]. Structural policy efforts must be undertaken to address these root causes.

Our study found that educational tools, such as motivation through participatory dialogue, in which advantages of getting a Pap test and building the behavioral confidence of minority women are attributed, when coupled with changes in the physical environment, such as availability and accessibility of Pap test, can contribute substantially to women’s ability to start getting the Pap test at regularly prescribed intervals. The concepts of value expectancy and self-efficacy, which are similar to participatory dialogue and behavioral confidence with subtle differences, have been used in the past for promoting cervical cancer screening among Hispanic American women [41,42]. The MTM constructs should be the foundational pillars of educational interventions. The advantages of getting a Pap test, such as early detection, having peace of mind for self and family, getting early treatment, and reduction in mortality due to cervical cancer, need to be underscored through educational interventions. The feelings of anxiety, physical discomfort/pain, invasion of modesty, embarrassment, and fear of misdiagnosis should be reduced in educational interventions through dialogue. Efforts to combat barriers in getting the Pap test must be undertaken to build the confidence of minority women through educational activities such as role-plays, psychodramas, or simulations. The construct of changes in the physical environment has not yet been used in the literature and our study lends support to the utilization of this dimension in future interventions. It is imperative that access and availability of Pap tests be increased for minority women at all locations in the U.S., especially for those without insurance and those who are indigent.

Our study found that in order to sustain the behavior of getting regular Pap tests, several correlates are essential. For sustenance of the behavior of getting regular Pap tests in both those who were not getting Pap tests and those who were getting Pap tests, all three constructs of MTM were significant and accounted for a substantial proportion of the variance. The construct of social support, which, in MTM, is more broadly referred to as changes in the social environment, has been used in the literature in the context of maintaining the behavior of getting Pap tests [39]. In many minority cultures, family and friends play an important role in the decision-making process for an individual. People are easily influenced by what others say or think of them. This aspect can be used as a potential advantage in health promotion interventions directed toward minority women for enhancing screening through Pap tests. Besides the traditional use of family, friends, and health professionals, in this regard, social media and advertisements are also gaining popularity and must be utilized more strategically in educational interventions. The constructs of emotional transformation and practice for change are unique to MTM and have not been tapped into in the past in the context of promoting the maintenance of Pap tests. Emotions or feelings are a powerful determinant of our behavior. If these can be harnessed into goals, then we can achieve our goals more effectively while combating self-doubt and fostering self-motivation. Educational interventions directed toward promoting Pap tests for minority women should facilitate participants to recognize their emotions, modulate these emotions, self-motivate themselves, and guide these emotions toward the goal of scheduling appointments with a physician and getting the Pap tests done on time. Likewise, a constant awareness of the importance of getting the Pap test done every three years can feed into building the practice for changing the construct of MTM.

While examining the socio-economic and healthcare access variables, as expected, less education, lack of health insurance, being unemployed, having less income, and not getting a recommendation from a healthcare provider were all significantly higher in the participants who did not receive the Pap test in the past three years. Influencing the socio-economic factors are under the purview of making structural policy changes but the lack of recommendation by healthcare providers is amendable through better education and training of healthcare providers that must be doggedly pursued.

### Strengths and Limitations

This study was the first to utilize a contemporary, fourth-generation Multi-theory Model in explaining the correlates of cervical cancer screening among minority women. The instrument used in the study had very good psychometric properties. The sample used in the study was nationally representative. The study had an adequate power and sample size to discern medium effect sizes. The study also provided support to MTM, an upcoming theoretical framework. However, there were some limitations to this study. First, the cross-sectional nature of the design precludes making temporal causal inferences between the independent and dependent variables. Future studies must utilize longitudinal experimental designs to provide more definitive evidence. Second, the study did not collect direct data in the form of medical records of Pap tests and relied only on self-reports, which are subject to biases. While some variables such as attitudes can only be measured through self-reports, future studies must utilize more objective data for variables that can have other means of measurement. Another limitation concerns the conducting of this survey only in English, which limited our sample to only those who could read and speak English. Future studies should offer other languages, e.g., Spanish, Chinese, Japanese, etc., to potentially capture more minority women. Finally, the study did not measure the stability of the instrument by test–retest reliability coefficients. Future studies, especially those undertaking interventional research, must test the stability of the instrument.

## 5. Conclusions

In the U.S., minority women have lower rates of cervical cancer screening through Pap tests. Efforts must be undertaken to increase these rates. The study identified contemporary theory-based correlates of cervical cancer screening among minority women using the framework of MTM and found that all constructs of this theory were significant predictors. The constructs of participatory dialogue, behavioral confidence, and changes in the physical environment explained a substantial proportion of the variance in starting the behavior of getting Pap tests, while the constructs of emotional transformation, practice for change, and changes in the social environment, along with lack of health insurance and annual household income of less than $25,000, significantly explained the likelihood to sustain the Pap test behavior of getting it every three years. Health promotion interventions based on MTM must be implemented to address the disparities of lower cervical cancer screenings among minority women.

## Figures and Tables

**Table 1:** Demographic characteristics of the sample (*N* = 364).

Variable	Categories	Participants Who Had Pap Smear 252, 69.2%	Participants Who Had Not Had Pap Smear 112, 30.8%	*p*-Value
Was the Pap smear normal	Yes	234 (93.0)	Not applicable	-
	No	18 (7.0)	Not applicable	-
Had hysterectomy	Yes	44 (17.5)	26 (23.2)	0.2
	No	208 (82.5)	86 (76.8)	
Age (M ± SD)	-	44.79 ± 13.3	44.1 ± 14.6	0.6
Hispanic or Latina	Yes	113 (44.8)	49 (43.8)	0.8
	No	139 (55.2)	63 (56.3)	
Religion	Christianity	178 (70.6)	70 (62.5)	0.1
	Non-Christianity	74 (29.4)	42 (37.5)	
Marital status	Married	105 (41.7)	34 (30.4)	0.2
	Never married	68 (27.0)	38 (33.9)	
	Divorced/Separated	41 (16.3)	24 (21.4)	
	Other	38 (15.1)	16 (14.3)	
Ethnicity	African American	93 (36.9)	38 (33.9)	0.2
	Hispanic–White	62 (24.6)	28 (25.0)	
	Asian	44 (17.5)	12 (10.7)	
	Others including multiethnic origin	53 (21.0)	34 (30.4)	
Comorbidities	Psychological	94 (37.3)	36 (32.1)	0.3
	Non-psychological	158 (62.7)	76 (67.9)	
Duration of U.S. residency (M ± SD)	-	39.7 ± 16.3	39.3 ± 16.5	0.8
Residence	Rural	49 (19.4)	15 (13.4)	0.2
	Suburban	105 (41.7)	44 (39.3)	
	Urban	98 (38.9)	53 (47.9)	
Encouraged Pap test by family/friends	Yes	107 (42.5)	43 (38.4)	0.5
	No	145 (57.5)	69 (61.6)	

**Table 2:** Socio-economic and healthcare access characteristics of the sample (*N =* 364).

Variable	Categories	Participants Who Had Pap Smear	Participants Who Had Not Had Pap Smear	*p*-Value
Education	Less than high school diploma	2 (0.8)	6 (5.4)	<0.001 *
	High school graduate	49 (19.4)	24 (21.4)	
	Some college but no degree	64 (25.4)	43 (38.4)	
	Associate/Bachelor	99 (39.3)	31 (27.7)	
	Graduate’s degree	38 (15.1)	8 (7.1)	
Healthcare insurance	Yes	239 (94.8)	80 (71.4)	<0.001 *
	No	13 (5.2)	32 (28.6)	
Employed	Yes	154 (61.1)	45 (40.2)	<0.001 *
	No	98 (38.9)	67 (59.8)	
Hours worked/week	-	35.7 ± 10.5	Not applicable	-
Income	<$25,000	50 (19.8)	31 (27.7)	0..004 *
	$25,000–$50,000	85 (33.7)	41 (36.6)	
	$50,001–$75,000	54 (21.4)	16 (14.3)	
	$75,001–$100,000	33 (13.1)	8 (7.1)	
	$100,001–$125,000	10 (4.0)	1 (0.9)	
	$125,001–$150,000	6 (2.4)	3 (2.7)	
	>$150,001	11 (4.4)	3 (2.7)	
Visited healthcare provider	Yes	210 (83.3)	63 (56.3)	0.2
	No	42 (16.7)	49 (43.8)	
Recommended Pap test by healthcare providers	Yes	147 (58.3)	35 (31.3)	<0.001 *
	No	105 (41.7)	77 (68.8)	

* Significant *p*-values.

**Table 3:** Comparison of Multi-theory Model (MTM) constructs of participants who had Pap test and those who did not have Pap test (*N =* 364).

MTM Construct	Had Pap Smear Test		*p*-Value
	Yes (*n =* 252)	No (*n* = 112)	
**Overall initiation score**	3.02 ± 0.99	1.69 ± 1.41	<0.001 *
**Subscales**			
Perceived advantages	15.62 ± 3.84	13.01 ± 5.65	<0.001 *
Perceived disadvantages	10.36 ± 4.55	10.99 ± 5.07	0.3
Participatory dialogue	5.55 ± 1.12	2.29 ± 146	<0.001 *
Behavioral confidence	14.01 ± 4.64	9.35 ± 5.53	<0.001 *
Changes in the physical environment	6.16 ± 1.85	4.59 ± 2.65	<0.001 *
**Overall sustenance**	2.98 ± 1.06	1.50 ± 1.34	<0.001 *
**Subscales**			
Emotional transformation	9.03 ± 2.85	5.31 ± 3.95	<0.001 *
Practice for change	8.68 ± 2.71	5.27 ± 4.02	<0.001 *
Changes in social environment	13.2 ± 4.65	8.18 ± 5.56	<0.001 *

* Significant *p*-values.

**Table 4:** Pearson correlations and reliability estimates for study variables in the sample population (*n =* 364).

Variables	1	2	3	4	5	6	7
1. Advantages	-	0.26 **	0.58 **	0.50 **	0.47 **	0.46 **	0.49 **
2. Disadvantages	0.26 **	1	0.04	0.05	−0.03	−0.06	−0.03
3. Behavioral confidence	0.58 **	0.04	1	0.75 **	0.81 **	0.78 **	0.72 **
4. Changes in the physical environment	0.50 **	0.05	0.75 **	1	0.73 **	0.73 **	0.64 **
5. Emotional transformation	0.47 **	−0.03	0.81 **	0.73 **	1	0.87 **	0.77 **
6. Practice for change	0.46 **	−0.07	0.79 **	0.73 **	0.87 **	1	0.82 **
7. Changes in social environment	0.49 **	−0.03	0.72 **	0.64 **	0.78 **	0.82 **	1
*Cronbach’s alpha values*	0.85	0.81	0.90	0.86	0.94	0.92	0.86

** *p* < 0.01. The Cronbach’s alpha value of the entire scale is 0.94.

**Table 5:** Multilevel modelling to predict likelihood for initiation of Pap test behavior among participants who had not had the Pap test over the past 3 years (*n =* 112).

Variables	Model 1		Model 2		Model 3		Model 4	
	B	*β*	B	*β*	B	*β*	B	*β*
**INITIATION MODEL**								
Constant	3.292 **	-	2.766 **	-	0.878 **	-	0.673 *	
**Socio-economic factors**								
Health insurance (ref: yes)	−0.899 **	−0.229	−0.696 **	−0.177	−0.275	−0.070	−0.276	−0.070
Employed (ref: yes)	−0.151	−0.058	−0.160	−0.062	−0.158	−0.061	−0.153	−0.059
Encouraged by HCW (ref: yes)	−0.313	−0.121	−0.211	−0.081	−0.132	−0.051	−0.113	−0.044
Income (ref: >$150,000)								
<$25,000	−0.263	−0.085	−0.135	−0.043	0.089	0.028	0.095	0.031
$25,000−$50,000	−0.498	−0.183	−0.401	−0.147	−0.158	−0.058	−0.150	−0.055
$50,001−$75,000	−0.197	−0.060	−0.098	−0.030	−0.074	−0.023	−0.038	−0.012
$75,001−$100,000	−0.429	−0.105	−0.139	−0.034	−0.034	−0.008	−0.055	−0.014
$100,001−$125,000	−0.697	−0.092	−0.560	−0.074	−0.293	−0.039	−0.327	−0.043
$125,001−$150,000	−0.207	−0.025	−0.170	−0.020	−0.204	−0.024	−0.219	−0.026
Participatory dialogue (advantages–disadvantages)	-	-	0.079 **	0.346	0.023 *	0.102	0.021 *	0.092
Behavioral confidence	-	-	-	-	0.149 **	0.617	0.117 **	0.484
Changes in the physical environment	-	-	-	-	-	-	0.106 *	0.184
R^2^	0.096	-	0.209	-	0.498	-	0.512	-
F	4.173 **	-	9.301 **	-	31.708 **	-	30.667 **	-
ΔR^2^	0.096	-	0.113	-	0.289	-	0.014	-
ΔF	4.173 **	-	50.236 **	-	202.64 **	-	10.149 *	-

* *p*-value < 0.05; ** *p*-value < 0.001; income variable was dummy-coded; and adjusted R^2^ = 0.495.

**Table 6:** Multilevel modelling to predict likelihood for sustenance of Pap test behavior among participants who had not had the Pap test over the past 3 years (*n =* 112).

Variables	Model 1		Model 2		Model 3		Model 4	
	B	*β*	B	*β*	B	*β*	B	*β*
**SUSTENANCE MODEL**								
Constant	2.847 **	-	0.046	-	−0.135	-	−0.216	-
**Socio-economic factors**								
Health insurance (ref: yes)	−1.018 **	−0.249	−0.458 **	−0.112	−0.365 *	−0.089	−0.353 *	−0.087
Employed (ref: yes)	−0.055	−0.020	0.053	0.020	0.056	0.021	0.070	0.026
Encouraged by HCW (ref: yes)	−0.388 *	−0.144	−0.138	−0.051	−0.128	−0.048	−0.093	−0.035
Income (ref: >$150,000)								
<$25,000	0.122	0.038	0.384 *	0.119	0.396 *	0.123	0.390 *	0.121
$25,000–$50,000	−0.071	−0.025	0.197	0.070	0.251	0.089	0.249	0.088
$50,001–$75,000	0.180	0.053	0.211	0.062	0.212	0.062	0.193	0.057
$75,001–$100,000	−0.037	−0.009	0.247	0.058	0.230	0.054	0.225	0.053
$100,001–$125,000	−0.267	−0.034	0.120	0.015	0.077	0.010	0.088	0.011
$125,001–$150,000	−0.008	−0.001	0.076	0.009	0.082	0.010	0.067	0.008
**MTM constructs**								
Emotional transformation	-	-	0.299 **	0.812	0.195 **	0.529	0.184 **	0.500
Practice for change	-	-	-	-	0.126 **	0.333	0.097 **	0.256
Changes in social environment	-	-	-	-	-	-	0.032 *	0.129
R^2^	0.097	-	0.714	-	0.740	-	0.745	-
F	4.247 **	-	88.00 **	-	90.998 **	-	85.338 **	-
ΔR^2^	0.097	-	0.616	-	0.026	-	0.005	-
ΔF	4.247	-	759.83 **	-	35.347 **	-	6.744 *	-

* *p*-value < 0.05; ** *p*-value < 0.001; income variable was dummy-coded; and adjusted R^2^ = 0.736.

**Table 7:** Multilevel modelling to predict likelihood for sustenance of Pap test behavior among participants who had the Pap test over the past 3 years (*n =* 252).

Variables	Model 1		Model 2		Model 3		Model 4	
	B	*β*	B	*β*	B	*β*	B	*β*
**SUSTENANCE MODEL**								
Constant	2.973 **	-	0.192	-	0.005	-	−0.037	-
**Socio-economic factors**								
Health insurance (ref: yes)	−0.674 *	−0.140	−0.423 *	−0.088	−0.436 *	−0.090	−0.398 *	−0.083
Employed (ref: yes)	0.235	0.107	0.123	0.056	0.120	0.055	0.141	0.065
Encouraged by HCW (ref: yes)	−0.066	−0.030	−0.126	−0.058	−0.114	−0.053	−0.090	−0.042
Income (ref: >$150,000)								
<$25,000	0.060	0.022	0.292	0.109	0.250	0.093	0.226	0.084
$25,000–$50,000	−0.151	−0.067	0.115	0.051	0.101	0.045	0.091	0.040
$50,001–$75,000	0.146	0.056	0.170	0.066	0.102	0.039	0.086	0.033
$75,001–$100,000	−0.003	−0.001	0.247	0.078	0.182	0.058	0.169	0.054
$100,001–$125,000	−0.447	−0.082	0.051	0.009	−0.053	−0.010	−0.044	−0.008
$125,001–$150,000	0.321	0.046	0.092	0.013	0.010	0.001	0.014	0.002
**MTM constructs**								
Emotional transformation	-	-	0.293 **	0.784	0.180 **	0.482	0.168 **	0.450
Practice for change	-	-	-	-	0.144 **	0.366	0.111 **	0.282
Changes in social environment	-	-	-	-	-	-	0.032 *	0.141
R^2^	0.052	-	0.645	-	0.687	-	0.694	-
F	1.479	-	43.849 **	-	47.952 **	-	45.254 **	-
ΔR^2^	0.052	-	0.593	-	0.042	-	0.007	-
ΔF	1.479	-	403.072 **	-	32.208 **	-	5.557 *	-

* *p*-value < 0.05; ** *p*-value < 0.001; income variable was dummy-coded; and Model 4 adjusted R^2^ = 0.833.

## Data Availability

The data presented in this study are available upon request from the corresponding author. The data are not publicly available due to ethical reasons.
